# 3-Carboxy­anilinium hemioxalate

**DOI:** 10.1107/S1600536809026427

**Published:** 2009-07-11

**Authors:** Lamia Bendjeddou, Sara Farah, Aouatef Cherouana

**Affiliations:** a Laboratoire de Chimie Moléculaire, du Contrôle, de l’Environnement et des Mesures Physico-Chimiques, Faculté des Sciences Exactes, Département de Chimie, Université Mentouri de Constantine, 25000 Constantine, Algeria

## Abstract

In the title compound, C_7_H_8_NO_2_^+^·0.5C_2_O_4_^2−^, the asymmetric unit consists of an 3-carboxy­anilinium cation, and one-half of an oxalate anion, which lies on a twofold rotation axis. The crystal packing is consolidated by inter­molecular N—H⋯O and O—H⋯O hydrogen bonds. The structure is built from infinite chains of cations and oxalate anions  extending parallel to the *b* and *c* axes. The crystal studied was a non-merohedral twin. The ratio of the twin components refined to 0.335 (3):0.665 (3).

## Related literature

Packing motifs, common patterns and hydrogen-bond networks in pure amino acids and in their crystals with organic acids are inter­esting for crystal engineering and for understanding structure–property relationships, see: Vijayan (1998 ▶); Nangia & Desiraju (1998 ▶); Desiraju (1997 ▶). For the structures of amino acid–carboxylic acid complexes, see: Bendjeddou *et al.* (2003 ▶); Cherouana *et al.* (2002 ▶). For bond-length data, see: Allen *et al.* (1987 ▶). For a description of the Cambridge Structural Database, see: Allen (2002 ▶). For graph-set motifs, see: Bernstein *et al.* (1995 ▶).
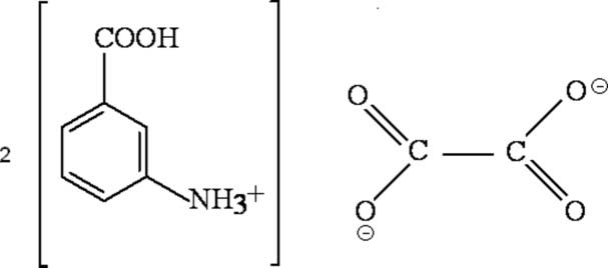


## Experimental

### 

#### Crystal data

C_7_H_8_NO_2_^+^·0.5C_2_O_4_^2−^
*M* *_r_* = 182.15
Monoclinic, 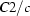

*a* = 22.034 (3) Å
*b* = 10.779 (2) Å
*c* = 6.9927 (10) Å
β = 103.918 (4)°
*V* = 1612.0 (4) Å^3^
*Z* = 8
Mo *K*α radiation
μ = 0.12 mm^−1^
*T* = 298 K
0.3 × 0.1 × 0.09 mm

#### Data collection

Nonius KappaCCD diffractometer
Absorption correction: none
8434 measured reflections
1836 independent reflections
1305 reflections with > 2σ
*R*_int_ = 0.056

#### Refinement

*R*[*F* ^2^ > 2σ(*F* ^2^)] = 0.050
*wR*(*F* ^2^) = 0.127
*S* = 1.02
1836 reflections
119 parameters
H-atom parameters constrained
Δρ_max_ = 0.23 e Å^−3^
Δρ_min_ = −0.30 e Å^−3^

### 

Data collection: *KappaCCD Reference Manual* (Nonius, 1998 ▶); cell refinement: *DENZO* and *SCALEPACK* (Otwinowski & Minor, 1997 ▶); data reduction: *DENZO* and *SCALEPACK*; program(s) used to solve structure: *SIR92* (Altomare *et al.*, 1993 ▶); program(s) used to refine structure: *SHELXL97* (Sheldrick, 2008 ▶); molecular graphics: *ORTEP-3* (Farrugia, 1997 ▶); software used to prepare material for publication: *WinGX* (Farrugia, 1999 ▶), *PARST97* (Nardelli, 1995 ▶), *Mercury* (Macrae *et al.*, 2006 ▶), *POVRay* (Persistence of Vision Team, 2004 ▶) and *PLATON* (Spek, 2009 ▶).

## Supplementary Material

Crystal structure: contains datablocks global, I. DOI: 10.1107/S1600536809026427/bx2219sup1.cif

Structure factors: contains datablocks I. DOI: 10.1107/S1600536809026427/bx2219Isup2.hkl

Additional supplementary materials:  crystallographic information; 3D view; checkCIF report

## Figures and Tables

**Table 1:** Hydrogen-bond geometry (Å, °)

*D*—H⋯*A*	*D*—H	H⋯*A*	*D*⋯*A*	*D*—H⋯*A*
O1*C*—H1*C*⋯O2^i^	0.82	1.75	2.560 (4)	169
N1—H1*N*⋯O1^ii^	0.89	1.92	2.798 (2)	169
N1—H2*N*⋯O2*C*^iii^	0.89	1.97	2.856 (2)	171
N1—H3*N*⋯O1	0.89	2.03	2.791 (2)	143

Symmetry codes: (i) 
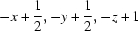
; (ii) 
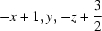
; (iii) 
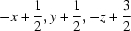
.

**Fig. 1.**
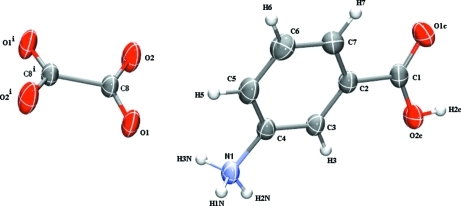
The cation and anion of (I), with the atom-labelling scheme. Displacement ellipsoids are drawn at the 50% probability level and H atoms are shown as spheres of arbitrary radii. Atoms with sufix (i) are generated by crystallographic rotation axis 2.

**Fig. 2.**
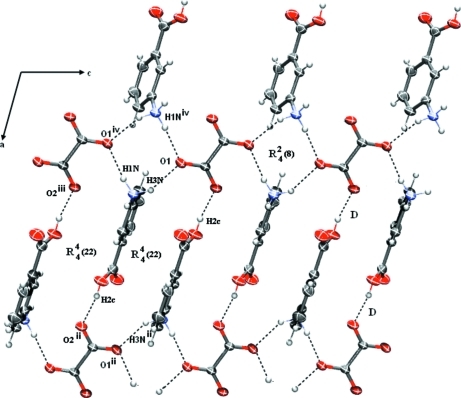
A view of the two-dimensional hydrogen-bonded network parallel to the (010) plane of (I), showing the aggregation of two hydrogen-bonding motifs, R12(5) and R44(24). Hydrogen bonds are drawn as dotted lines. Atoms marked with (ii), (iii) and (iv) are at the symmetry positions (1/2 - x,1/2 - y, 1 - z), (x, y, 1 + z), (1 - x, y, 1.5 - z) respectively.

**Fig. 3.**
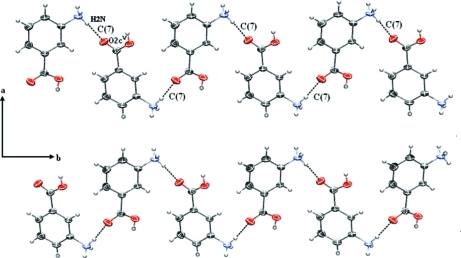
Projection down the a axis of the lattice of C7H8NO2+. 0.5 C2 O42-, showing the formation of C(7) chains along [010]. Atom marked with (v) is at symmetry positions (1/2 - x, 1/2 + y, 1.5 - z).

## supplementary crystallographic information

### Comment

A comparative study of the packing motifs, common patterns and the hydrogen-bond networks in crystals of pure amino acids and in their crystals with organic acids is interesting for crystal engineering and for understanding structure-property relation ships (Vijayan (1998), Nangia & Desiraju (1998), Desiraju (1997)). Amino acids crystallize easily with organic acids in general and with oxalic acid in particular. These systems are interesting as molecular materials which exhibit nonlinear optical properties.

The present study, which reports the crystal structure of 3-carboxyanilinium acid with oxalic acid, (I), forms part of a series of X-ray investigations being carried out in our laboratory on amino acid-carboxylic acid complexes. The X-ray investigations on these complexes have revealed interesting and useful data regarding the ionization states of individual molecules, their stoichiometry and intermolecular aggregation patterns (Bendjeddou *et al.*, 2003, Cherouana *et al.*, 2002).

Fig. 1 shows the molecular structure of (I). The amino acid molecule exists in the cationic form with a positively charged amino group and uncharged carboxylic acid group. The oxalate anion is flat and completely deprotonated and lies across a crystallographic rotation axis 2. The bond lengths and angles are all normal for their types (Allen *et al.*, 1987).

In the title compound the ions are connected *via* a three-dimensional N—H···O and O—H···O hydrogen bonds network (Table 1). Unexpectedly, there are no centrosymmetric hydrogen bonded dimers between the carboxylic acid groups of adjacent 3-carboxyanilinium cations which is a characteristic feature found in most salts of 3- and 4-aminobenzoic acid (Cambridge Structural Database, Version 5.29; Allen, 2002). All ammonium H atoms are involved in hydrogen bonds with two different anions and one cation. Two of these interactions link the anions and cations in an alternating fashion into extended rings along the [001] direction, which can be described by the graph-set motif *R*^2^_1_(5) (Bernstein *et al.*, 1995). The combination of each N—H1N···O1 and N—H3N···O1 hydrogen bonds, with the only O—H···O which is a finit chain with a D(4) motif, generates two centrosymmetric fused rings a long [001] direction which can be described by the graph-set motif *R*^4^_4_ (22) (Fig.2). The third interaction link the cations with the carbonyl O atom into zigzag chains along the [010] direction, which can be described by the graph-set motif C(7) (Fig.3).

### Experimental

Brown needle-shaped single crystals of (I) were grown from a saturated aqueous solution containing *m*-aminobenzoic and oxalic acid in a 2:1 stoichiometric ratio.

### Refinement

All H atoms attached to C, N and O atom were fixed geometrically and treated as riding with C—H = 0.93 Å, N—H = 0.89Å and O—H = 0.82 Å with *U*_iso_(H) = 1.2*U*_eq_(C,*N*) or *U*_iso_(H) = 1.5*U*_eq_(O).

Owing to the initial poor refinement, the search for the possibility of a non-merohedral twinning was carried out using the TwinRotMat procedure within *PLATON* (Spek, 2009). The crystal appears to be twinned about (1 0 0) with the rotation matrix: 1 0 1.516 0 - 1 0 0 0 - 1 The ratio of the two twin components was refined to 0.335 (3):0.665 (3).

### Crystal data

**Table:**

C_7_H_8_NO_2_^+^·0.5C_2_O_4_^2^^−^	*F*(000) = 760
*M**_r_* = 182.15	*D*_x_ = 1.501 Mg m^−^^3^
Monoclinic, *C*2/*c*	Mo *K*α radiation, λ = 0.71073 Å
Hall symbol: -C 2yc	Cell parameters from 6947 reflections
*a* = 22.034 (3) Å	θ = 1.0–27.5°
*b* = 10.779 (2) Å	µ = 0.12 mm^−^^1^
*c* = 6.9927 (10) Å	*T* = 298 K
β = 103.918 (4)°	Prism, brown
*V* = 1612.0 (4) Å^3^	0.3 × 0.1 × 0.09 mm
*Z* = 8	

### Data collection

**Table:**

Nonius KappaCCD diffractometer	1305 reflections with > 2σ
Radiation source: fine-focus sealed tube	*R*_int_ = 0.056
graphite	θ_max_ = 27.5°, θ_min_ = 5.1°
ω scans	*h* = −28→27
8434 measured reflections	*k* = −14→14
1836 independent reflections	*l* = −8→9

### Refinement

**Table:**

Refinement on *F*^2^	0 restraints
Least-squares matrix: full	H-atom parameters constrained
*R*[*F*^2^ > 2σ(*F*^2^)] = 0.050	*w* = 1/[σ^2^(*F*_o_^2^) + (0.06*P*)^2^ + 0.8696*P*] where *P* = (*F*_o_^2^ + 2*F*_c_^2^)/3
*wR*(*F*^2^) = 0.127	(Δ/σ)_max_ < 0.001
*S* = 1.02	Δρ_max_ = 0.23 e Å^−^^3^
1836 reflections	Δρ_min_ = −0.30 e Å^−^^3^
119 parameters	

### Special details

**Table:**

Geometry. All e.s.d.'s (except the e.s.d. in the dihedral angle between two l.s. planes) are estimated using the full covariance matrix. The cell e.s.d.'s are taken into account individually in the estimation of e.s.d.'s in distances, angles and torsion angles; correlations between e.s.d.'s in cell parameters are only used when they are defined by crystal symmetry. An approximate (isotropic) treatment of cell e.s.d.'s is used for estimating e.s.d.'s involving l.s. planes.

### Fractional atomic coordinates and isotropic or equivalent isotropic displacement parameters (Å^2^)

**Table:**

	*x*	*y*	*z*	*U*_iso_*/*U*_eq_	
O1C	0.19114 (6)	0.20422 (15)	0.7825 (2)	0.0416 (4)	
H1C	0.1543	0.1925	0.7822	0.062*	
O2C	0.18039 (7)	0.02007 (15)	0.6332 (3)	0.0454 (4)	
O1	0.47841 (6)	0.34729 (17)	0.4737 (2)	0.0444 (5)	
O2	0.41945 (6)	0.32562 (19)	0.1690 (2)	0.0511 (5)	
N1	0.40615 (7)	0.35616 (16)	0.7510 (2)	0.0311 (4)	
H1N	0.4405	0.3469	0.8473	0.037*	
H2N	0.3819	0.4139	0.7851	0.037*	
H3N	0.4167	0.3793	0.6412	0.037*	
C2	0.27904 (9)	0.1208 (2)	0.6919 (3)	0.0298 (5)	
C6	0.37088 (10)	0.0240 (2)	0.6326 (3)	0.0419 (6)	
H6	0.3912	−0.0462	0.6022	0.050*	
C7	0.30879 (10)	0.0167 (2)	0.6429 (3)	0.0363 (5)	
H7	0.2873	−0.0580	0.6169	0.044*	
C3	0.31077 (8)	0.2324 (2)	0.7322 (3)	0.0286 (5)	
H3	0.2911	0.3018	0.7686	0.034*	
C4	0.37232 (8)	0.2388 (2)	0.7174 (3)	0.0283 (5)	
C8	0.47040 (9)	0.3362 (2)	0.2924 (3)	0.0299 (5)	
C5	0.40230 (10)	0.1354 (2)	0.6675 (3)	0.0378 (5)	
H5	0.4435	0.1410	0.6575	0.045*	
C1	0.21199 (9)	0.1102 (2)	0.7004 (3)	0.0321 (5)	

### Atomic displacement parameters (Å^2^)

**Table:**

	*U*^11^	*U*^22^	*U*^33^	*U*^12^	*U*^13^	*U*^23^
O1C	0.0238 (7)	0.0494 (10)	0.0560 (10)	−0.0059 (6)	0.0181 (7)	−0.0094 (8)
O2C	0.0338 (8)	0.0464 (10)	0.0578 (10)	−0.0126 (7)	0.0145 (8)	−0.0077 (8)
O1	0.0245 (7)	0.0812 (13)	0.0303 (8)	−0.0043 (7)	0.0121 (6)	−0.0032 (8)
O2	0.0201 (7)	0.0984 (15)	0.0351 (8)	−0.0040 (8)	0.0073 (6)	−0.0016 (9)
N1	0.0200 (8)	0.0438 (11)	0.0302 (9)	−0.0009 (7)	0.0075 (7)	0.0004 (8)
C2	0.0244 (10)	0.0378 (12)	0.0278 (10)	−0.0007 (8)	0.0072 (8)	0.0020 (9)
C6	0.0366 (12)	0.0437 (14)	0.0483 (13)	0.0077 (10)	0.0159 (11)	−0.0037 (11)
C7	0.0367 (11)	0.0375 (13)	0.0363 (11)	−0.0045 (10)	0.0119 (9)	−0.0022 (10)
C3	0.0231 (9)	0.0363 (12)	0.0276 (10)	0.0022 (8)	0.0082 (8)	0.0002 (9)
C4	0.0218 (10)	0.0389 (12)	0.0237 (9)	−0.0017 (8)	0.0045 (7)	0.0014 (8)
C8	0.0208 (10)	0.0406 (12)	0.0298 (10)	0.0004 (8)	0.0091 (8)	0.0002 (9)
C5	0.0234 (10)	0.0525 (15)	0.0393 (12)	0.0020 (10)	0.0113 (9)	−0.0004 (11)
C1	0.0269 (10)	0.0400 (13)	0.0299 (10)	−0.0040 (9)	0.0076 (8)	0.0058 (10)

### Geometric parameters (Å, °)

**Table:**

O1C—C1	1.302 (3)	C2—C1	1.497 (3)
O1C—H1C	0.8200	C6—C5	1.378 (3)
O2C—C1	1.222 (2)	C6—C7	1.389 (3)
O1—C8	1.244 (3)	C6—H6	0.9300
O2—C8	1.245 (2)	C7—H7	0.9300
N1—C4	1.459 (3)	C3—C4	1.386 (3)
N1—H1N	0.8900	C3—H3	0.9300
N1—H2N	0.8900	C4—C5	1.382 (3)
N1—H3N	0.8900	C8—C8^i^	1.557 (4)
C2—C7	1.384 (3)	C5—H5	0.9300
C2—C3	1.385 (3)		
			
C1—O1C—H1C	109.5	C2—C3—C4	118.88 (19)
C4—N1—H1N	109.5	C2—C3—H3	120.6
C4—N1—H2N	109.5	C4—C3—H3	120.6
H1N—N1—H2N	109.5	C5—C4—C3	120.95 (19)
C4—N1—H3N	109.5	C5—C4—N1	118.88 (17)
H1N—N1—H3N	109.5	C3—C4—N1	120.16 (18)
H2N—N1—H3N	109.5	O1—C8—O2	126.74 (19)
C7—C2—C3	120.55 (18)	O1—C8—C8^i^	117.5 (2)
C7—C2—C1	118.60 (19)	O2—C8—C8^i^	115.8 (2)
C3—C2—C1	120.85 (18)	C6—C5—C4	119.77 (19)
C5—C6—C7	120.0 (2)	C6—C5—H5	120.1
C5—C6—H6	120.0	C4—C5—H5	120.1
C7—C6—H6	120.0	O2C—C1—O1C	124.04 (19)
C2—C7—C6	119.9 (2)	O2C—C1—C2	121.5 (2)
C2—C7—H7	120.1	O1C—C1—C2	114.49 (17)
C6—C7—H7	120.1		
			
O1C—C1—C2—C3	−12.3 (3)	C2—C3—C4—C5	−1.3 (3)
O1C—C1—C2—C7	167.71 (18)	N1—C4—C5—C6	−179.12 (19)
O2C—C1—C2—C3	167.2 (2)	C3—C4—C5—C6	−0.3 (3)
O2C—C1—C2—C7	−12.9 (3)	C4—C5—C6—C7	1.6 (3)
C1—C2—C3—C4	−178.32 (19)	C5—C6—C7—C2	−1.2 (3)
C7—C2—C3—C4	1.7 (3)	O1—C8—C8^i^—O1^i^	−167.6 (2)
C1—C2—C7—C6	179.58 (19)	O1—C8—C8^i^—O2^i^	12.1 (3)
C3—C2—C7—C6	−0.4 (3)	O2—C8—C8^i^—O1^i^	12.1 (3)
C2—C3—C4—N1	177.46 (19)	O2—C8—C8^i^—O2^i^	−168.3 (2)

Symmetry codes: (i) −*x*+1, *y*, −*z*+1/2.

### Hydrogen-bond geometry (Å, °)

**Table:**

*D*—H···*A*	*D*—H	H···*A*	*D*···*A*	*D*—H···*A*
O1C—H1C···O2^ii^	0.82	1.75	2.560 (4)	169
N1—H1N···O1^iii^	0.89	1.92	2.798 (2)	169
N1—H2N···O2C^iv^	0.89	1.97	2.856 (2)	171
N1—H3N···O1	0.89	2.03	2.791 (2)	143

Symmetry codes: (ii) −*x*+1/2, −*y*+1/2, −*z*+1; (iii) −*x*+1, *y*, −*z*+3/2; (iv) −*x*+1/2, *y*+1/2, −*z*+3/2.
